# GnT-V-mediated aberrant N-glycosylation of TIMP-1 promotes diabetic retinopathy progression

**DOI:** 10.1007/s11033-024-09388-7

**Published:** 2024-03-18

**Authors:** Xiaoting Xi, Yanni Yang, Qianbo Chen, Jia Ma, Xuewei Wang, Yachun Deng, Xi Wang, Yan Li

**Affiliations:** 1 Ophthalmology Department, The First Affiliated Hospital of Kunming Medical University, No. 295, Xichang Road, Kunming, 650032 Yunnan China; 2 Ophthalmology Department, The Second Hospital of Ningbo, Ningbo, 315010 Zhejiang China

**Keywords:** N-glycosylation, Diabetic retinopathy, N-acetylglucosaminyltransferase V, Tissue inhibitor of metalloproteinase 1, Vascular endothelial growth factor

## Abstract

**Background:**

Vascular endothelial growth factor (VEGF) signaling pathway plays an important role in the progression of diabetic retinopathy (DR). The glycosylation modification process of many key functional proteins in DR patients is abnormal. However, the potential involvement of abnormal N-glycoproteins in DR progression remains unclear.

**Methods:**

Glycoproteomic profiling of the vitreous humor was performed. The level of protein and N-glycoprotein was confirmed by Western blot and Lectin blot, respectively. The cell viability and migration efficiency were detected by CCK-8 and Transwell assay. Flow cytometry was conducted to analyze the level of cell apoptosis and reactive oxygen specie. Malondialdehyde, superoxide dismutase activity and VEGF content were detected by Enzyme linked immunosorbent assays. The interaction of metalloproteinase 1 (TIMP-1) with N-acetylglucosamine transferase V (GnT-V) was detected by GST pull-down. Hematoxylin and eosin staining and choroidal and retinal flat mount stained with fluorescein isothiocyanate-Dextran assay were used for functional research in vivo.

**Results:**

We found that N-glycosylation was up-regulated in DR rats and high glucose (HG)-induced human retinal pigment epithelium cell line ARPE-19. HG-induced inhibited the viability of ARPE-19 cells and promoted cell apoptosis and oxidative stress (OS), but these effects were reversed with kifunensine treatment, GnT-V knockdown and TIMP-1 mutation. Additionally, GnT-V binds to TIMP-1 to promote N-glycosylation of TIMP-1. Over-expression of GnT-V inhibited the viability of ARPE-19 cells and promoted cell apoptosis, OS and VEGF release, which these effects were reversed with TIMP-1 mutation. Interestingly, over-expression of GnT-V promoted retinal microvascular endothelial cells (RMECs) angiogenesis but was revered with TIMP-1 mutation, which was terminally boosted by VEGF-A treatment. Finally, knockdown of GnT-V relieved DR progression.

**Conclusion:**

The findings indicate that GnT-V can promote RMECs angiogenesis and ARPE-19 cells injury through activation VEGF signaling pathway by increasing TIMP-1 N-glycosylation level, which provides a new theoretical basis for the prevention of DR.

## Introduction

Diabetic retinopathy (DR) is a microvascular complication of diabetes mellitus (DM), can cause blindness in severe cases. The prevalence rate of DM is in the fast-growing stage by counting of The International Diabetes Federation [1]. In 2040, DM cases will be 642 million in the world, and China’s DM cases have reached 109 million [1]. China will become the largest number of DM patients country in the world [1]. The pathogenesis of DR is influenced by multiple factors, including environment, genes and signaling pathways. However, the mechanism has not yet been fully clarified. Retinal pigment epithelial (RPE) cells constitute the blood-retinal barrier and play an important role in the pathogenesis of DR [2] HG environment can destroy the polar structure of RPE cells, damage the blood-retinal barrier, and promote the secretion of vascular endothelial growth factor (VEGF) and neovascularization, above final factors lead to the loss of vision [3, 4]. VEGF-receptor 2 (VEGF-R2) is mainly located on the basal side or lateral lumen of retinal microvascular endothelial cells (RMECs), and RMECs, as blood retinal internal barrier, play an important role in DR retinal microvascular regeneration [5]. It is found that many factors and proteins are secreted by RPE cells and can promote retinal angiogenesis by binding to the surface membrane receptors of RMECs [6]. However, the mechanism of angiogenesis of RMECs was induced by VEGF from RPE cells secret in the HG environment needs to be further explored.

Previous studies have shown that many protein post-translational modifications (PTMs) play important role in regulating immunity and blood vessels by modifying the related protein molecules and activating pathways, which is one of the epigenetic mechanisms of diabetes and its complications, including protein glycosylation [7, 8]. Glycosylation is closely related to many important life activities and diseases [9–11]. Glycosylation modification is mainly divided into two types: N-glycosylation and O-glycosylation. Most glycoproteins contain only one glycosylation type. In recent years, most of the studies on glycosylation in DR focus on O-glycosylation, and there are few studies on N-glycosylation [12–14]. Increasing glucose level or over-expression of transferase increased the production of uridine diphosphate N-acetylglucosamine resulting in intracellular protein glycosylation up-regulation. However, it is unclear which protein glycosylation plays an important role in DR.

The tissue inhibitor of metalloproteinase (TIMP) family is an endogenous inhibitor of matrix metalloproteinase. Studies have found that the interaction of metalloproteinase 1 (TIMP-1) is up-regulated in vitreous cavity fluid of proliferative DR patients, and the up-regulation of TIMP-1 may be a sign of the beginning and development of angiogenesis [20, 21], and TIMP-1 increase with development and progression of DR [15]. TIMPs glycosylation is associated with malignant biological behavior and angiogenesis of cancer cells [16]. TIMP-1 and VEGF are the main factors of angiogenesis, but the relationship between TIMP-1 and VEGF has not been reported in DR. Therefore, exploring the mechanism of TIMP-1 regulating VEGF will provide a new perspective and theoretical basis for DR prevention and treatment. In this study, we found that N-acetylglucosaminyltransferase V (GnT-V) can promote RMECs angiogenesis and ARPE-19 cells injury through activation VEGF signaling pathway by increasing TIMP-1 N-glycosylation level.

## Materials and methods

### Patients

Vitreous cavity fluid samples were collected from 22 patients. These patients included macular hole patients (MH group, n = 6), diabetic patients with macular hole but without DR (DM + MH group, n = 4), DR group (n = 6), and intravitreal anti-VEGF injection in patients with DR (DR + anti-VEGF group, n = 6).

### Vitreous cavity fluid extraction

A 25 G vitrectomy head was used to cut 0.5 ml vitreous humor in the center of the vitreous cavity, and then the vitreous cavity fluid was quickly added to an eppendorf tube and stored in a − 80 °C freezer. The specific operation is carried out according to the method of Zhang et al. [17].

### Protein extraction and digestion

The samples were removed from − 80 °C, centrifuged at 12,000×*g* for 10 min at 4 °C after thawing, and the supernatant was transferred to a new centrifuge tube. proteins were extracted using a protein extraction kit (Thermo Fisher).

The extracted protein solution was digested by alkylation with 5 mM dithiothreitol and 11 mM iodoacetamide, and protein samples were diluted with TEAB. Then 2% trypsin was added for overnight digestion and finally 1% trypsin for 4 h.

### LC–MS/MS analysis

The PTM was enriched, then desalted to obtain supernatant containing glycopeptide, and then lyophilized for liquid chromatography–tandem mass spectrometry (LC–MS/MS) analysis. The peptides were loaded onto a home-made reversed-phase analytical column (15-cm length, 75 μm i.d.) after dissolved in solvent A (0.1% formic acid). The total flow rate was 400 nl/min on an EASY-nLC 1000 UPLC system with an increase from 6 to 23% solvent B (0.1% formic acid in 98% acetonitrile) over 26 min, 23 to 35% in 8 min and climbing to 80% in 3 min then holding at 80% for the last 3 min.

Peptides were processed by NSI source, and then analyzed by tandem mass spectrometry in Q exact vetm Plus (Thermo) connected to UPLC, and the LC–MS/MS data was processed by Maxquant search engine (v.1.5.2.8). Tandem mass spectra were searched in human uniprot database concatenated with reverse decoy database. Trypsin/P was designated as a cleavage enzyme that allows up to four deletions to be cleaved.The mass tolerance of precursor ions was set to 20 ppm in the first search, 5 ppm in the main search, and 0.02 Da for fragment ions.Carbamoyl methyl on Cys is designated as fixed modification, acetylation modification and oxidation on Met are designated as variable modification. Deamidation with ^18^O(N) and NQ were designated as variable modifications for the identification of N-glycopeptides. The false discovery rate for peptide and protein identification was adjusted to < 1%, and the lowest fraction of modified peptide was set to > 40.

### Motif analysis

Sequence models of modified 21-mers in all protein sequences were analyzed by the Soft MoMo algorithm.

### Functional enrichment

Genome was analyzed as a whole, the differential proteins were detected by Fisher method, and the types of differential proteins were enriched by GO; Perform KEGG pathway analysis on differential proteins to analyze the pathways in which these proteins are annotated; the InterPro domain database aligns the gene and protein sequences to analyze the structural domains and functional sites of the protein.

### Western blot analysis

Proteins were separated by SDS-PAGE gel electrophoresis followed by transfer and blocking with skim milk, The blocked proteins were then incubated overnight at 4 °C with primary antibodies for gp90phox, Nrf2, NQO1, GnT-V, VEGF-A, and LTL lectin (Vector Laboratories, Burlingham, CA) purchased from Abcam, and GAPDH was used as the internal reference control. Next, horseradish peroxidase was added to the membrane and incubated for 2 h at room temperature. The protein was then visualized with the ECL Western Blot Detection Kit (USA), and the band intensities were determined with ImageJ software.

### Cell transfection

The cDNA fragment of GnT-V was amplified and cloned into pcDNA3.1 vector to construct oe-GnT-V overexpression vector. The vector or si-GnT-V (siRNA) (RiboBio, Guangzhou, China) was transfected into ARPE-19 (ATCC) cells with Lipofectamine® 2000 (Invitrogen; Thermo Fisher Scientific, Inc.) and 48 h later, the transfection efficiency was measured by western blot analysis.

### Cell viability and apoptosis measurement

Cell viability was detected with CCK-8 kit (AbMole, USA), absorbance value was measured at 450 nm wavelength, and cell proliferation ability was calculated.

ARPE-19 cells were treated with Annexin V-FITC Apoptosis Detection Kit (Beyotime, China), and cell apoptosis was detected by flow cytometry.

### Enzyme linked immunosorbent assays

Malondialdehyde (MDA) and superoxide dismutase (SOD) activities were measured using Enzyme linked immunosorbent assays (ELISA) kits purchased from Jiancheng Bioengineering Institute, and VEGF levels were detected using ELISA kits purchased from Abcam.

### Reactive oxygen species analysis

Reactive oxygen species (ROS) rates were measured with a ROS detection kit (ThermoFisher Scientific), and ROS levels were analyzed by flow cytometry.

### Immunofluorescence

Cell samples were permeabilized with PBS containing 0.5% TritonX-100 and 5% goat serum for 15 min at room temperature. Then, incubated with rabbit antibody, Alexa Fluor conjugated secondary antibody with GnT-V (1:50, #ab177941, Abcam) and VEGF-A (1:200 dilution, #ab52917, Abcam) for 1 h, 0.1% DAPI staining 5 min under Nikon Eclipse 80i microscope.

### CRISPR-Cas9-mediated gene disruption

To establish a TIMP-1 deficient cell model, the TIMP-1 CRISPR/Cas9 KO plasmid was purchased from RiboBio (Guangzhou, China) and transfected into ARPE-19 for 48 h, after which positive cells were isolated and cultured. TIMP-1 knockout clones were isolated by single-cell dilution cloning method and identified by western blot. At the same time, the CRISPR/Cas9 double nickase plasmid was used as a negative control.

### Lentiviral packaging experiment

For TIMP-1-WT and TIMP-1-2NQ recombination in TIMP-1-deficient ARPE-19 cells, the packaging plasmids combined with pCDH-TIMP-1-WT-Flag (m) or pCDH-TIMP-1-2NQ-Flag (m) was co-transfected into HEK293T cells, viral particles were collected at 48 h, and cells were further infected with viral particles for 24 h, and finally positive stable cell lines were selected with puromycin. Finally, TIMP-1 recombinant clones were isolated from the positive population by single-cell dilution cloning method.

### GST-pull down assays

The GST pull-down assay was performed according to the research method of Zhang et al. [18]. The pGEX-GST-TIMP-1 and pcDNA-Myc-GnT-V plasmids were transformed into BL21 cells and HEK293T cells, respectively, to generate GST-TIMP-1 protein and Myc-GnT-V protein, respectively and the Pierce™ GST kit (Pierce; Thermo Fisher Scientific, Inc.) was used for detection, and protein samples were evaluated by western blot.

### Transwell assays

Cells were subjected to migration assays using the Transwell system (Millipore, Bedford, Massachusetts, USA). Cells in each chamber were photographed with a Nikon Eclipse 80i microscope, and the number of migrating cells was counted with a microscope (Olympus, Tokyo, Japan) in three random fields.

### Tube formation assay

RMECs were seeded on Matrigel-coated Ibitreat angiogenesis slides (Germany), and angiogenesis was observed and recorded under a microscope 6 h later.

### Animal model and treatment

Male SD rats (weight: 220–250 g) were purchased from the Experimental Animal Center of Kunming Medical University, and housed in an environment of alternating light and dark at 22 °C and 50% humidity, given ad libitum food and water. The DR rat model was constructed with streptozotocin (STZ) according to Xu et al. [19]. Rats were divided into the following groups, control group (CK), DR group, DR + si-GnT-V group (DR rats receiving si-GnT-V treatment group). Eight weeks after STZ injection, all rats were anesthetized by intracavitary injection and euthanised.

### Hematoxylin and eosin (H&E) staining

The eyeballs were fixed with paraformaldehyde and embedded in paraffin, cut into 5 μm thick, stained with HE solution. The pictures were observed under the microscope.

### Choroidal and retinal flat mount stained with fluorescein isothiocyanate-dextran

The rats were anesthetized and fluorescein isothiocyanate-Dextran (FITC-D) (Sigma-Aldrich Corp, USA) was injected into the tail vein. Retinas and eyecups were isolated and tiled on glass slides, and fluorescence images were captured with a fluorescence microscope (Nikon). The specific operation method is carried out according to the previously reported scheme [20].

### Protein–protein docking

Three-dimensional structures of GnT-V and TIMP-1 were downloaded from the RCSB Protein Data Bank (www.rcsb.org). Docking GnT-V and TIMP-1 were performed using ZDOCK server (http://zdock.umassmed.edu/). The Analyze Protein Interface of Discovery Studio (DS) 2019 was used to analyze and map the interaction mode of protein–protein complex [21].

### Statistical analysis

Data statistics and analysis were performed using GraphPad Prism 8 and expressed as mean ± standard deviation. Student’s t-test was used to analyze differences between groups, and one-way ANOVA and Tukey’s test were used to analyze differences between multiple groups, with statistical significance at P < 0.05, and all experiments were performed with three biological replicates.

## Results

### N-glycosylation up-regulation was closely to DR

**Fig. 1:**
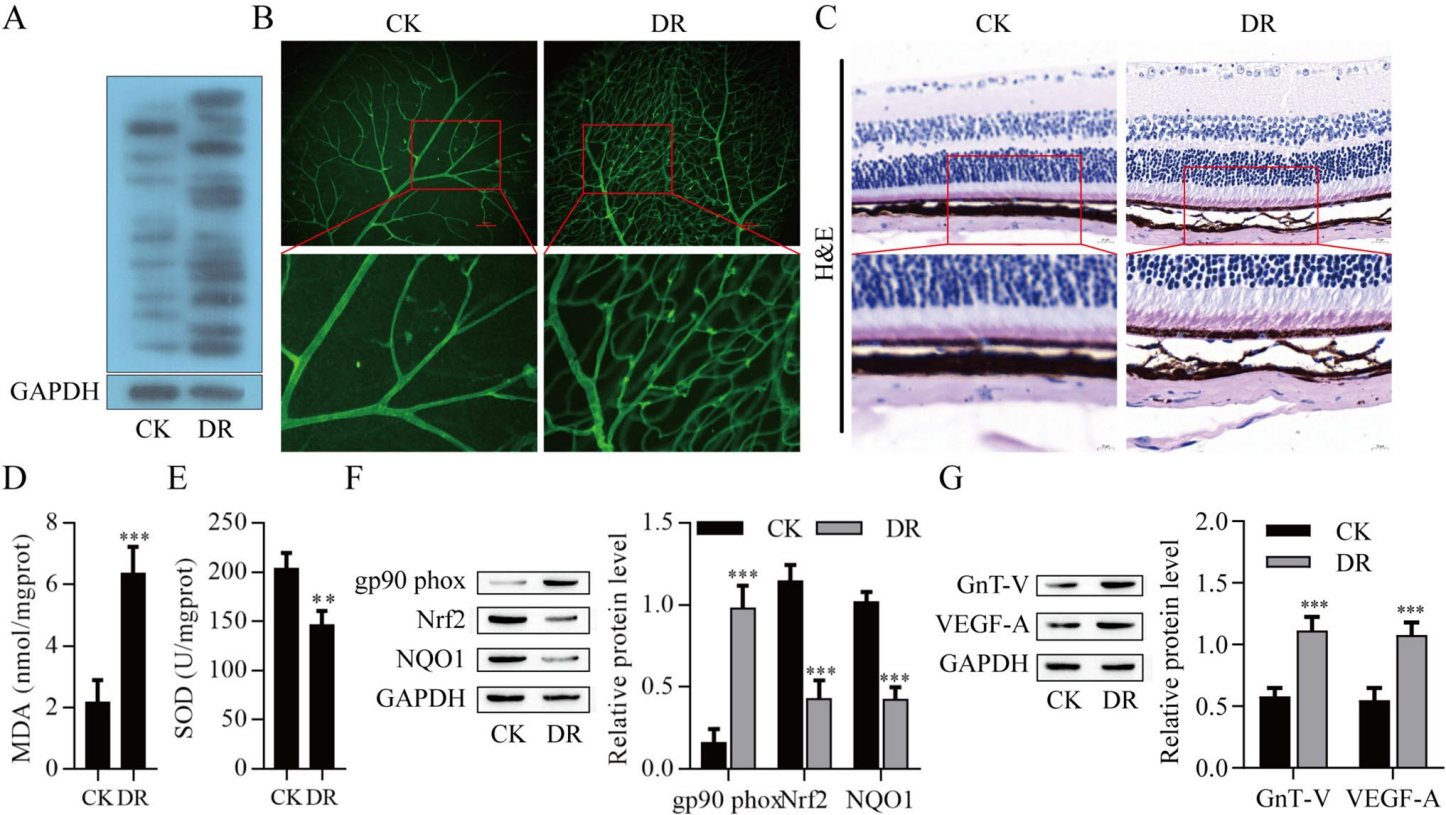
N-glycosylation up-regulation was closely to DR. **A** Lectin blot analysis of DR rat retina. **B** Representative images of flat-mounted choroid retina. Scale bar = 100 µm. **C** Result after staining with hematoxylin and eosin. Scale bar = 10 µm. **D** MDA content. **E** SOD activity. **F**–**G** Western blot analysis of retinal protein expression of gp90phox, Nrf2, NQO1, GnT-V and VEGF-A. **P* < *0.01,***P < 0.001 vs.CK. *CK* normal group control, *DR* model group

Considering the critical role of N-glycosylation in DR, the level of N-glycosylation on DR will be explored. Indeed, we observed significantly N-glycosylation up-regulation in the whole retina with DR rats compared with control retinas (Fig. 1A). When FITC-D was perfused on the choroid and retina of CK rats, retinal blood vessels showed a uniform and orderly network structure. However, the retina of DR rats showed prominent spherical diseased blood vessels, representing retinal neovascular vesicles, while the choroid of DR rats had a large number of disordered blood vessel aggregation, representing choroidal neovascular clusters (Fig. 1B). As shown in H&E staining, in the control group, the structures of retinal and choroidal layers were clear and orderly, and the RPE cells were closely arranged, but in the DR group, the thickness of RPE cell layer decreased, the structure was loose, and the number of cells decreased (Fig. 1C). The MDA content (an oxidative stress marker) and SOD activity (and anti-oxidative enzyme) were significantly elevated and reduced, respectively, in the whole retina with DR group compared with in control retinas (Fig. 1D, E). Additionally, DR group significantly increased gp90^phox^ level (an NADPH oxidase 2 contributing to oxidative stress) level compared with control retinas and decreased the oxidative stress inhibitors level of Nrf2 and NQO1 (Fig. 1F). As shown in Fig. 1, DR increased the N-glycosylation level. Thus, we identified the protein expression of GnT-V, a glycosyltransferase, was significantly increased in the DR group. More importantly, the DR group also remarkably elevated the level of VEGF-A (Fig. 1G). These observations suggest that N-glycosylation up-regulation is closely related to DR and may play a role in this process.

### High glucose induced N-glycosylation level and injury of ARPE-19 cells

**Fig. 2:**
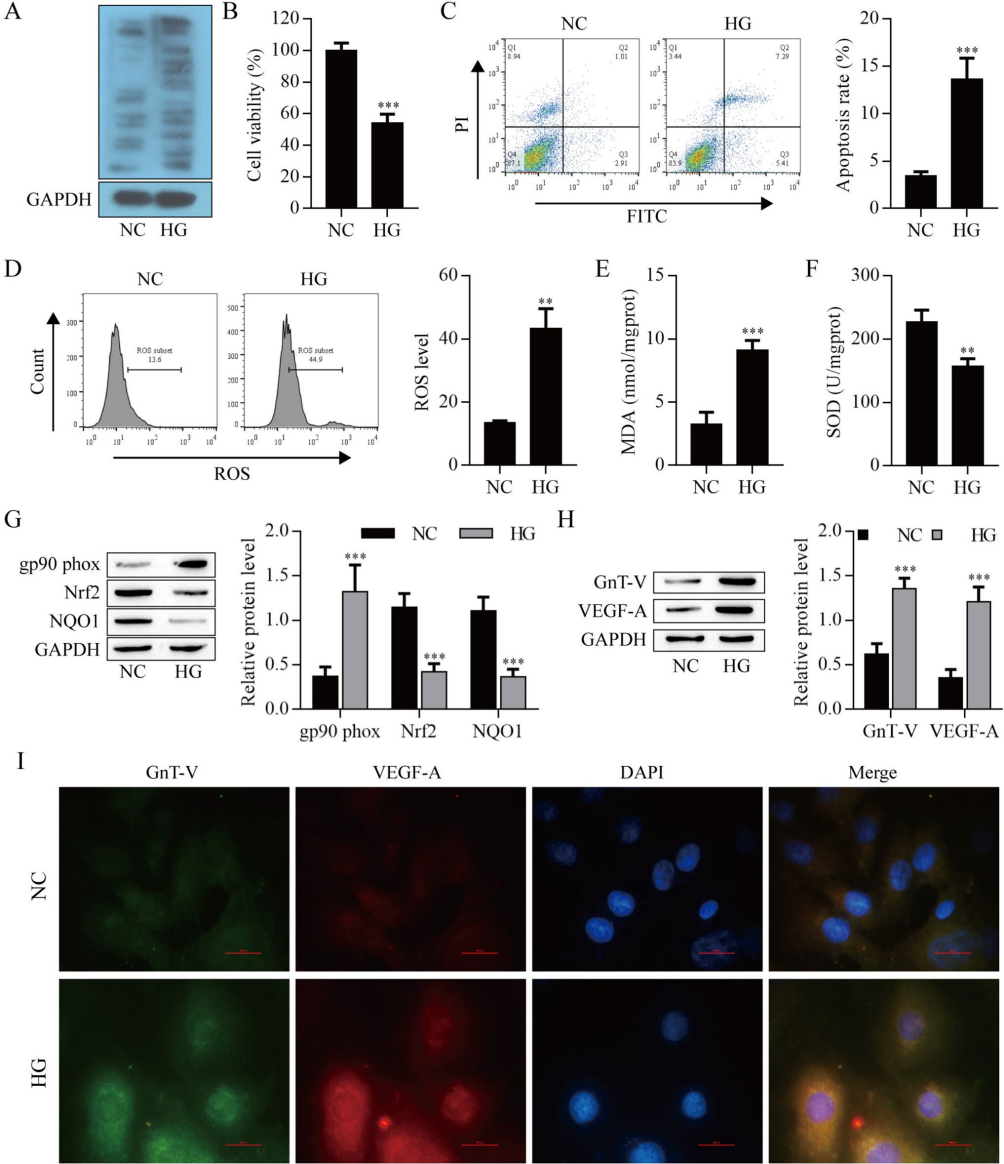
High glucose induced N-glycosylation level and injury of ARPE-19 cells. **A** Lectin blot analysis of ARPE-19 cells. **B** ARPE-19 cell viability was detected by CCK8. Apoptosis **C** and ROS **D** of ARPE-19 cells were detected by flow cytometry. **E** MDA content. **F** SOD activity. **G**, **H** Analysis of the expression levels of gp90phox, Nrf2, NQO1 proteins and GnT-V, VEGF-A proteins in ARPE-19 cells by Western blot (I) Immunofluorescence analysis of GnT-V and VEGF-A protein levels. **P < 0.01 and ***P < 0.001 vs. NC. *HG* high glucose group, *NC* negative control

Considering the critical role of RPE cells in DR, we constructed a DR cell model by HG-induced ARPE-19 cells. As shown in Fig. 2A, HG-induced increased N-glycosylation level by using Lectin blot. HG-induced inhibited the viability in ARPE-19 cells (Fig. 2B) and elevated the apoptosis and ROS level (Fig. 2C, D). As shown in Fig. 2E and F, the content of MDA was increased, while the activity of SOD was decreased in the HG-induced ARPE-19 cells. Furthermore, the expression of gp90^phox^ was significantly elevated following HG induction. In contrast, the protein level of Nrf2 and NQO1 showed reduced expression (Fig. 2G). We also measured the GnT-V and VEGF-A proteins level by Western blot and immunofluorescence, HG-induced increased GnT-V and VEGF-A proteins level (Fig. 2H, I). These data indicate that HG induced N-glycosylation and injury of ARPE-19 cells.

### N-glycosylation was required for HG-induced ARPE-19 cells injury

**Fig. 3:**
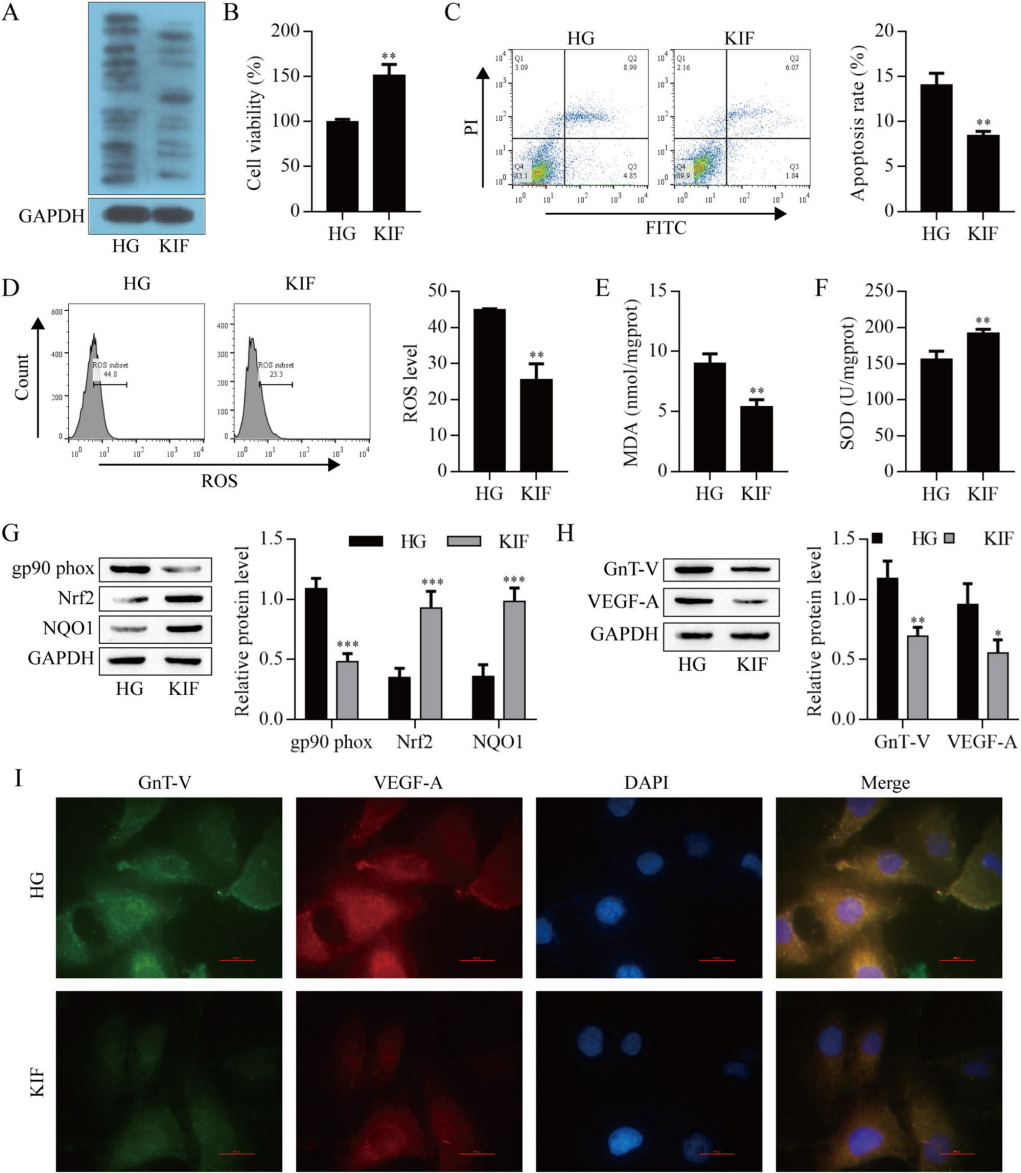
Analysis of the role of N-glycosylation in HG-induced ARPE-19 cells damage. **A** Lectin blot analysis of ARPE-19 cells. **B** The viability of ARPE-19 cells was detected by CCK-8 assay. Apoptosis **C** and ROS **D** of ARPE-19 cells were detected by flow cytometry. **E** MDA content. **F** SOD activity. **G** Western blot analysis of gp90^phox^, Nrf2 and NQO1 protein levels in ARPE-19 cells. Western blot (**H**) and immunofluorescence (**I**) analysis of GnT-V and VEGF-A protein levels in ARPE-19 cells. *P < 0.05, **P < 0.01 and ***P < 0.001 vs. HG. *HG* high glucose group, *KIF* kifunensine group

To determine the role of N-glycosylation in HG-induced ARPE-19 cells injury, we used kifunensine (KIF, an inhibitor of key enzymes participating in N-glycosylation). As shown in Fig. 3A, KIF inhibited the N-glycosylation level. KIF up-regulated cell viability (Fig. 3B), and down-regulated apoptosis and ROS levels (Fig. 3C,D). KIF significantly reduced MDA content (Fig. 3E) and elevated SOD activity (Fig. 3F). Additionally, KIF significantly decreased gp90^phox^ level and increased the level of Nrf2 and NQO1 (Fig. 3G). More importantly, KIF also remarkably reduced the level of GnT-V and VEGF-A (Fig. 3H,I). Overall, inhibition of N-glycosylation contributed to the protective effects of HG-induced ARPE-19 cells injury.

### GnT-V knockdown attenuates HG-induced damage in ARPE-19 cells

**Fig. 4:**
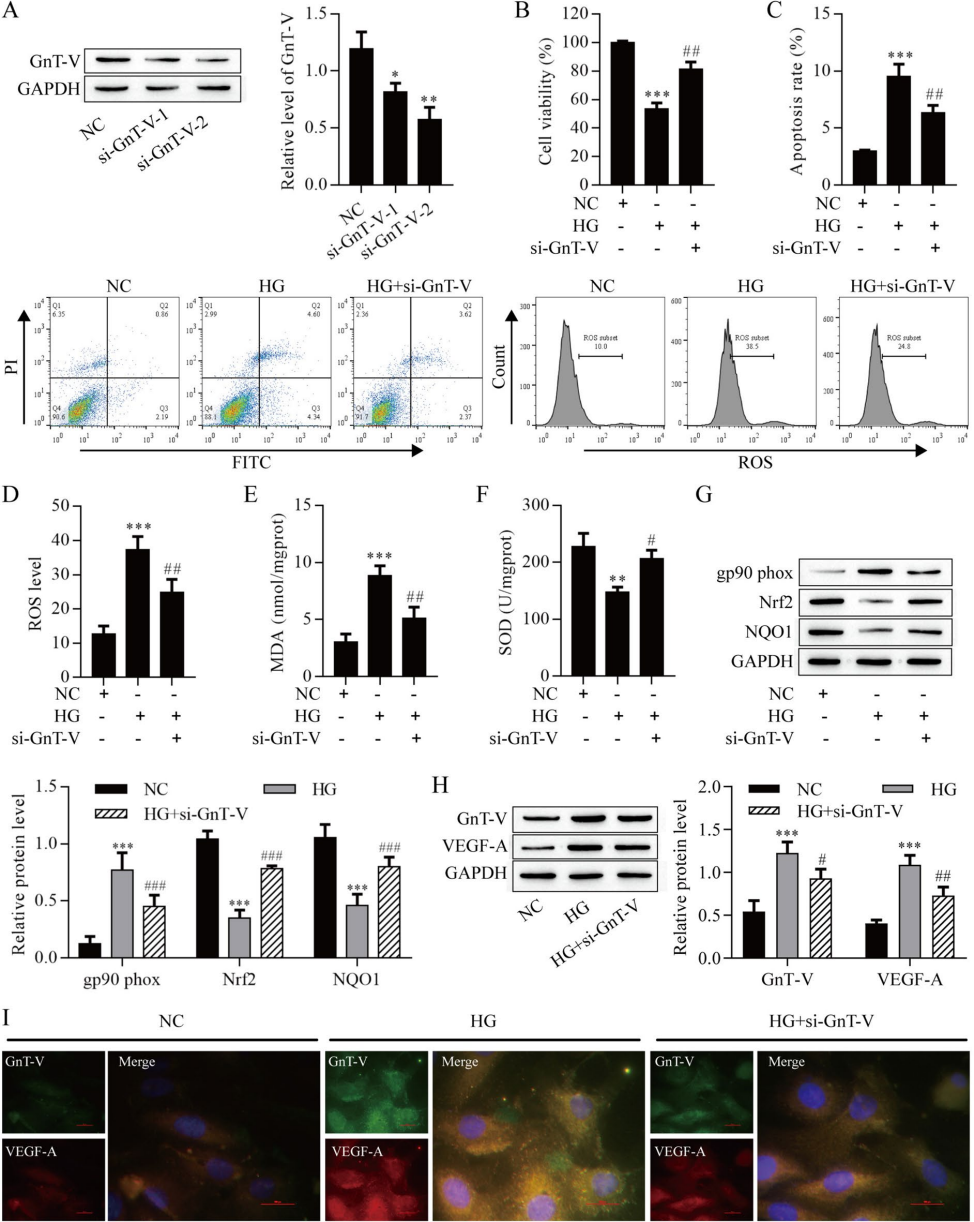
Analysis of the effect of GnT-V knockdown on HG-induced ARPE-19 cell damage. **A** Western blot analysis of GnT-V protein levels in ARPE-19 cells. **B** ARPE-19 cell viability was detected by CCK8 assay. Apoptosis **C** and ROS **D** of ARPE-19 cells were detected by flow cytometry. **E** MDA content. **F** SOD activity. **G** Western blot analysis of gp90^phox^, Nrf2 and NQO1 protein levels in ARPE-19 cells. Western blot (**H**) and immunofluorescence (**I**) analysis of GnT-V and VEGF-A protein levels in ARPE-19 cells. *P < 0.05, **P < 0.01 and ***P < 0.001 vs. the NC group; ^#^P < 0.05, ^##^P < 0.01 and ^###^P < 0.001 vs. the HG group. *NC* negative control, *HG* high glucose group, *si* small interfering RNA group

To investigate the relevance of GnT-V in HG-induced injury of ARPE-19 cells, ARPE-19 cells were transfected with siRNA. Western blot results showed that GnT-V protein expression level was significantly reduced (Fig. 4A). In addition, the GnT-V depletion significantly decreased the inhibitory effect of HG-induced on ARPE-19 cells viability (Fig. 4B). On the contrary, the levels of apoptosis and ROS were elevated after HG-induced but reduced with GnT-V knockdown (Fig. 4C, D). The content of MDA was elevated after HG-induced, and GnT-V knockdown remarkably decreased the MDA content (Fig. 4E). On the contrary, SOD activity was reduced after HG-induced but increased with GnT-V knockdown (Fig. 4F). In addition, the expression of gp90^phox^ was increased after HG-induced but reduced with si-GnT-V treatment. The expression of Nrf2 and NQO1 were decreased after HG-induced, whereas knockdown of GnT-V remarkably elevated the expression of Nrf2 and NQO1 (Fig. 4G). Western blot and immunofluorescence results showed that HG-induced increased the expression of GnT-V and VEGF-A, whereas knockdown of GnT-V remarkably decreased the expression of GnT-V and VEGF-A (Fig. 4H, I). These data indicated that GnT-V plays a critical role in the HG-induced ARPE-19 cells injury.

### Differentially expressed N-glycosylation sites with N-glycoproteins in vitreous humor

**Fig. 5:**
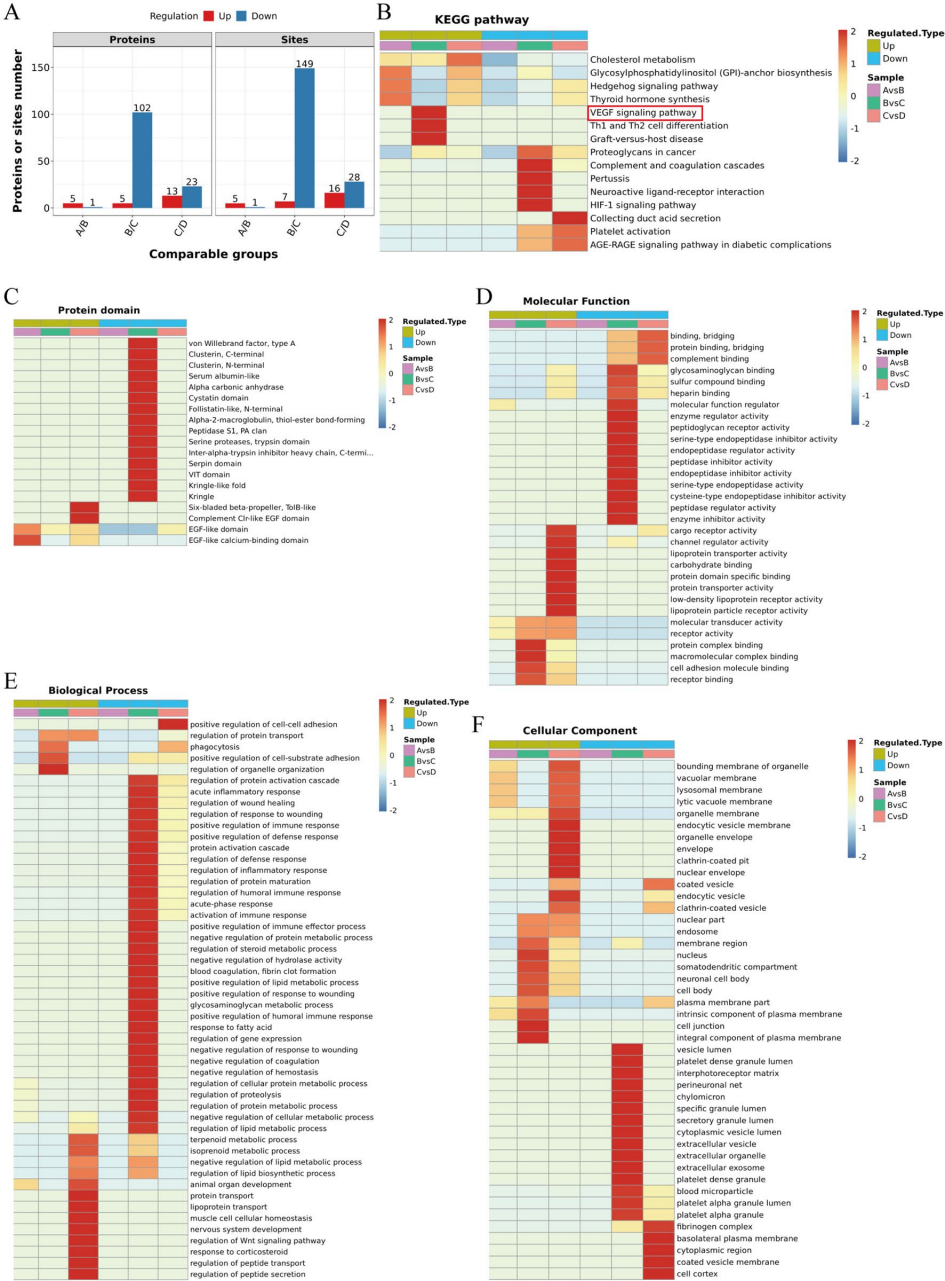
Cluster analysis. **A** The number of N-glycoproteins with N-glycosylation in vitreous cavity fluid. **B** KEGG pathway analysis. **C** Domain analysis. **D** function analysis. **E** Bioinformatics analysis **F** Cellular component analysis. KEGG, Encyclopedia of Genes and Genomes. **A**, macular hole patients (MH group, n = 6); **B**, diabetic patients with macular hole but without DR (DM + MH group, n = 4); **C**, diabetic retinopathy (DR group, n = 6); **D**, intravitreal anti-VEGF injection in patients with DR (DR + anti-VEGF group, n = 6)

A total of 6 N-glycoproteins with 6 N-glycosylation sites exhibited a significantly different expression pattern in MH and DM + MH. Compared with DM + MH, 107 N-glycoproteins with 156 N-glycosylation sites showed a significantly different expression pattern in DR. Compared with DR, 36 N-glycoproteins with 44 N-glycosylation sites exhibited a significantly different expression pattern in DR + anti-VEGF (Fig. 5A).

### Clustering analysis of N-glycoproteins

The protein cluster analysis is shown in Fig. 5B–F. KEGG enrichment results show that proteins undergoing glycosylation are enriched into several important pathways (Fig. 5B). In MH compared with DM + MH, the pathways of glycosylphosphatidylinositol (GPI)-anchor biosynthesis, hedgehog and thyroid hormone synthesis were enriched in the up-regulated proteins. Compared with the DR group, the expression of VEGF signaling pathway, Th1 and Th2 cell differentiation and graft-versus-host disease were up-regulated in the DM + MH group; Complement and coagulation cascades, pertussis, neuroactive ligand-receptor down-regulated proteins are mainly enriched in the secretory pathway. In DR compared with DR + anti-VEGF, the pathways of cholesterol metabolism and GPI-anchor biosynthesis were enriched in the up-regulated proteins; Collecting duct acid secretion, platelet activation and AGE-RAGE are enriched in signaling pathways of diabetic complications.

Domain analysis of the protein after glycosylation showed that, EGF-like and EGF-like calcium-binding domains are more abundant compared to DM + MH; no cluster was obtained in the down-regulated region. In DM + MH compared with DR, no cluster was obtained in the up-regulated region; The domains of von willebrand factor (type A), clusterin (C-terminal), clusterin (N-terminal), serum albumin-like, alpha carbonic anhydrase, cystain, follistatin-like, alpha-2-macroglobulin, peptidase S1 (PA clan), serine proteases (trypsin), serpin, VIT, kringle and kringle-like fold were enriched in the down-regulated proteins. In DR compared with DR + anti-VEGF, the domains of six-bladed beta-propeller (ToIB-like) and complement Clr-like EGF were in the up-regulated region; no cluster was obtained in the down-regulated region (Fig. 5C).

In MH compared with DM + MH, as few differential proteins, no cluster was obtained in the up-regulated region. In DM + MH compared with DR and DR compared with DR + anti-VEGF, for the differential proteins, it was found that they were enriched in Fig. 5D–F.

These results might provide important information on the need for this vital pathway as VEGF signaling pathway in DR. Looking at the N-glycosylation modified differential proteins enriched in the VEGF signal pathway, the N-glycosylation levels of CP, TIMP-1, DKK3, Serpina3 and APOB increased significantly. Among them, the N-glycosylation level of TIMP-1 protein increased by 3.85 times in DR compared with DM + MH (Table 1).

**Table 1:** N-glycosylation modified differential proteins enriched in VEGF signal pathway

Protein accession	Position	DR/DM + MH Ratio	Regulated Type	DR/DM + MH P value	Gene name
P00450	397	1.3726	Up	0.0222	CP
P01033	101	3.8530	Up	0.0249	TIMP1
Q9UBP4	106	2.7119	Up	0.0000	DKK3
P01011	186	2.5538	Up	0.0013	Serpina3
P04114	1523	1.5287	Up	0.0002	APOB

*DR* diabetic retinopathy, *DM + MH* diabetic patients with macular hole but without DR, *CP* ceruloplasmin, *TIMP1* tissue inhibitor of metalloproteinase 1, *DKK3* dickkopf-related protein 3, *Serpina3* Alpha-1-antichymotrypsin, *APOB* apolipoprotein B

### GnT-V-mediated TIMP-1 N-glycosylation was important in HG-induced ARPE-19 cells injury

**Fig. 6:**
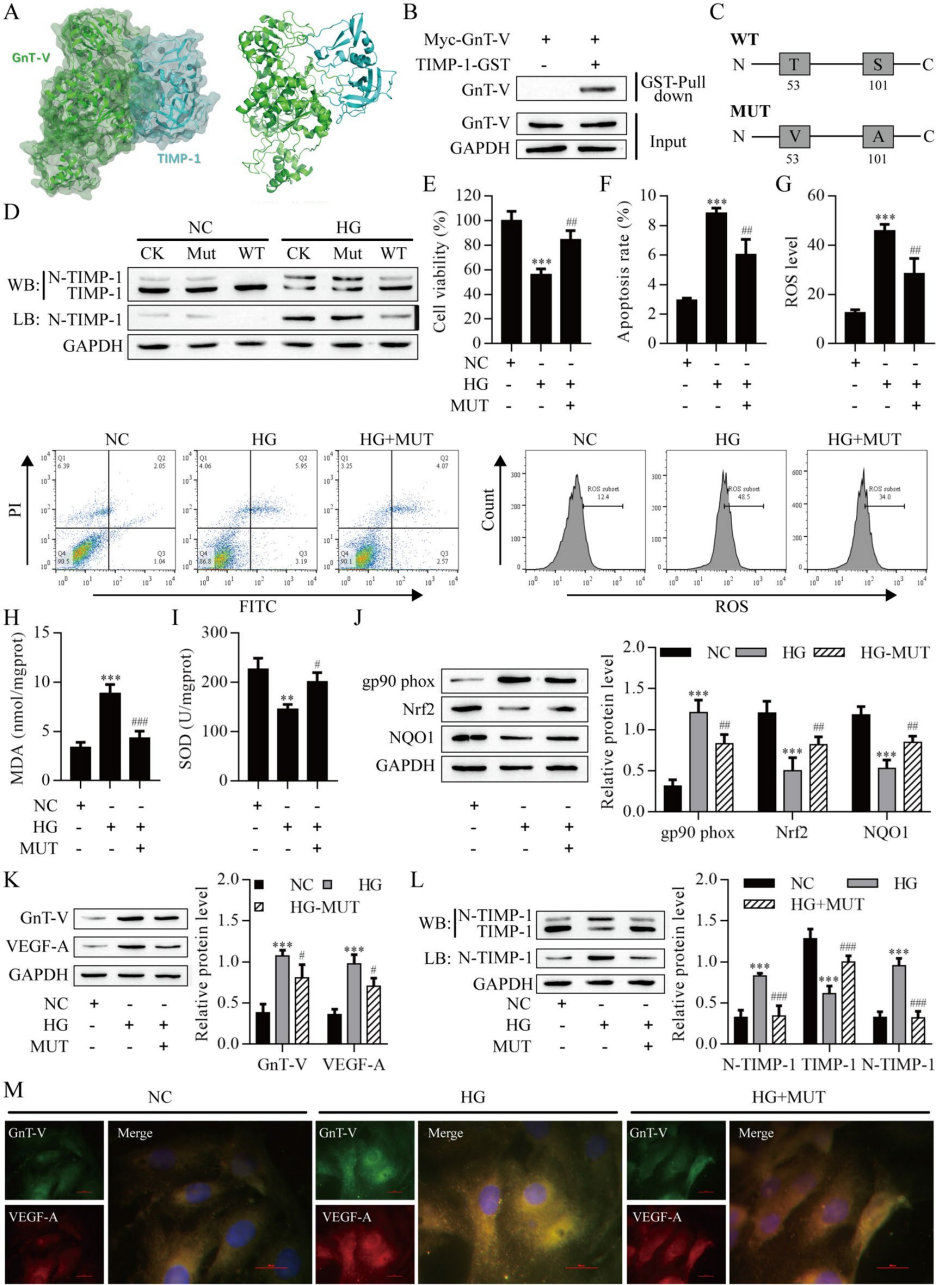
GnT-V-mediated TIMP-1 N-glycosylation was important in HG-induced ARPE-19 cells injury. **A** GnT-V/TIMP-1 exhibited surface complementarity. **B** GST pull-down assay. **C** Schematic diagram of TIMP-1 mutants. **D** Western blot and Lectin blot measured N-glycosylation of TIMP-1. **E** The viability of ARPE-19 cells was detected by CCK8 assay. Apoptosis **F** and ROS **G** of ARPE-19 cells were detected by flow cytometry. **H** MDA content. **I** SOD activity. **J** Western blot analysis of gp90^phox^, Nrf2 and NQO1 protein levels in ARPE-19 cells. Western blot (**K)** and immunofluorescence (**M)** analysis of GnT-V and VEGF-A protein levels in ARPE-19 cells. **L** Western blot and Lectin blot measured N-glycosylation of TIMP-1. **P* < *0.01 and ***P < 0.001 vs. the NC group; ^#^P < 0.05, ^##^P < 0.01 and ^###^P < 0.001 vs. the HG group. *NC*, blank control; *HG*, high glucose; *WT*, wild type; *MUT*, mutation

Using the DS 2016 ZDOCK program, the GnT-V/TIMP-1 binding pose in 3D space was calculated and evaluated. The ZDOCK docking results indicated that GnT-V/TIMP-1 exhibited surface complementarity in the interface area (Fig. 6A). To further determine whether the interaction between GnT-V and TIMP-1 was direct, GST pull-down assays were performed using the TIMP-1-GST protein expressed in and purified from bacteria. As shown in Fig. 6B GST-fused TIMP-1 pulled down GnT-V. These data suggest that GnT-V binds to TIMP-1.

We then depleted TIMP-1 using specific single-guide RNAs in ARPE-19 cells to construct a TIMP-1-deficient cell model, and then the constructs of TIMP-1-WT and a mutant form TIMP-1-MUT were stably added back. The results showed that TIMP-1 N-glycosylation was completely ablated in TIMP-1-MUT mutant cells (Fig. 6C). When we used HG to treat CK, TIMP-1-WT and -MUT cells, N-glycosylation of TIMP-1 was significantly increased in the CK and TIMP-1-WT cells compared with NC groups, but a few increased in the TIMP-1-MUT cells (Fig. 6D). To determine whether N-glycosylation of TIMP-1 regulates HG-induced RPE cells injury in vitro. CCK-8 results showed that TIMP-1-MUT significantly increased viability in HG-induced ARPE-19 cells (Fig. 6E). Flow cytometry analysis showed that apoptosis and ROS levels were remarkably decreased in TIMP-1-MUT ARPE-19 cells than in HG-induced ARPE-19 cells (Fig. 6F, G). The content of MDA was elevated after HG-induced, and TIMP-1-MUT remarkably decreased the MDA content (Fig. 6H). On the contrary, SOD activity was reduced after HG-induced but increased with TIMP-1-MUT (Fig. 6I). The expression of gp90^phox^ was increased after HG-induced but reduced with TIMP-1-MUT treatment. The expression of Nrf2 and NQO1 were decreased after HG-induced, whereas mutation of TIMP-1 remarkably elevated the expression of Nrf2 and NQO1(Fig. 6J). Western blot and immunofluorescence results showed that HG-induced increased the expression of GnT-V and VEGF-A, whereas mutation of TIMP-1 remarkably decreased the expression of GnT-V and VEGF-A (Fig. 6K, M). We further validated whether TIMP-1 was N-glycosylated at HG-induced ARPE-19 cells. We observed that N-glycosylation of TIMP-1 was completely promoted by HG-induced, but mutation of TIMP-1 reversed the promoting effect (Fig. 6L). Together, the results indicated that N-glycosylation of TIMP-1 plays a critical role in the injury of HG-induced ARPE-19 cells.

### GnT-V overexpression caused ARPE-19 cells injury by promoting N-glycosylation of TIMP-1

**Fig. 7:**
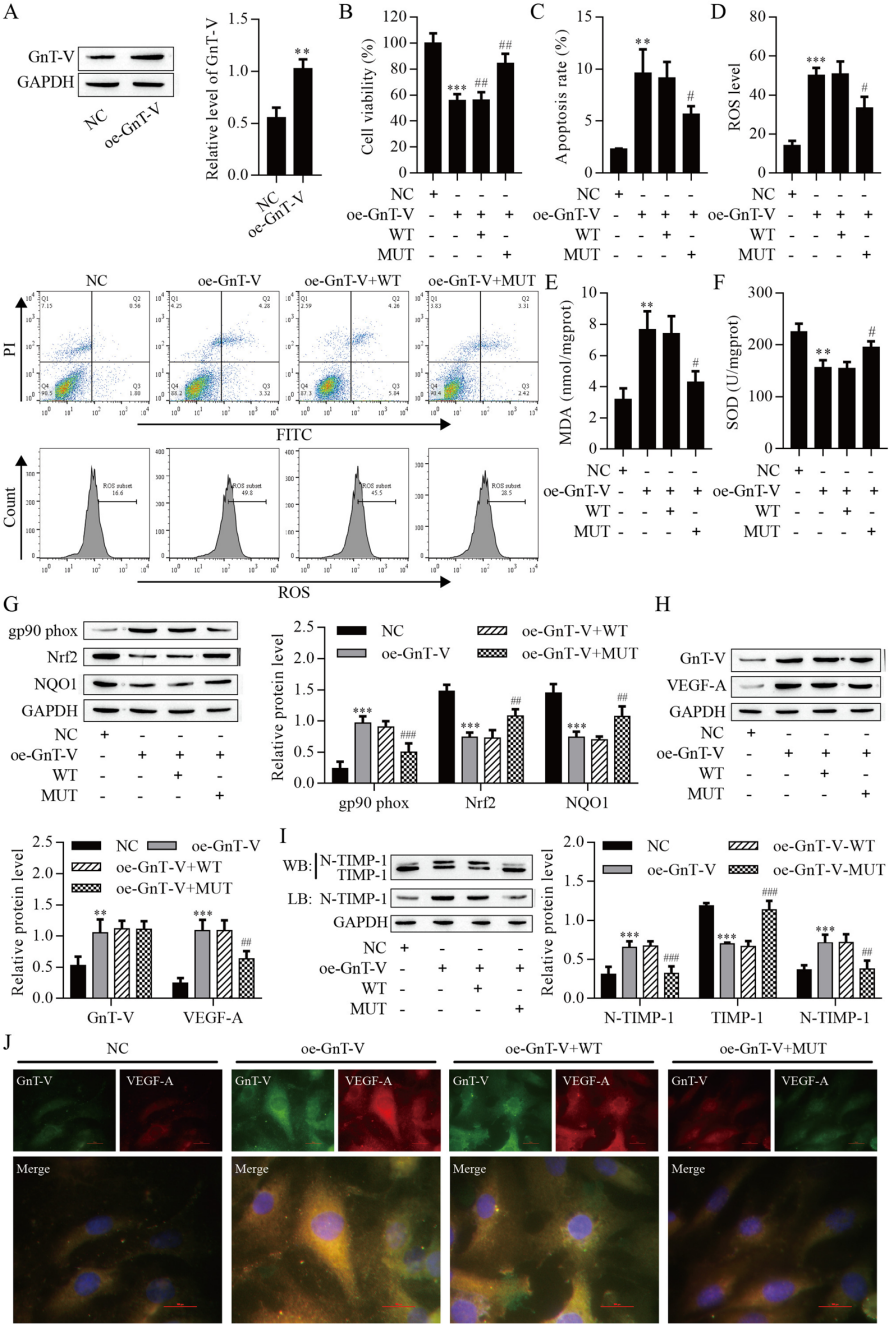
GnT-V over-expression caused ARPE-19 cells injury by promoting N-glycosylation of TIMP-1. **A** Western blot analysis of GnT-V protein levels in ARPE-19 cells. **B** The viability of ARPE-19 cells was detected by CCK8 assay. Apoptosis **C** and ROS **D** of ARPE-19 cells were detected by flow cytometry. **E** MDA content. **F** SOD activity. **G** Western blot analysis of gp90^phox^, Nrf2 and NQO1 protein levels in ARPE-19 cells. Western blot (**H)** and immunofluorescence (**J)** analysis of GnT-V and VEGF-A protein levels in ARPE-19 cells. **I** Western blot and Lectin blot measured N-glycosylation of TIMP-1. **P < 0.01 and ***P < 0.001 vs. the NC group; ^#^P < 0.05, ^##^P < 0.01 and ^###^P < 0.001 vs. the oe-GnT-V group. *NC* blank control, *oe* over-expression, *MUT* mutation, *WT* wild type

Furthermore, the effect of the GnT-V/TIMP-1 axis on the ARPE-19 cells was explored. The ARPE-19 cells were transfected with GnT-V over-expression plasmid to up-regulation GnT-V expression. Western blot results displayed that the GnT-V protein level was significantly increased (Fig. 7A). Notably, GnT-V over-expression inhibited the viability of ARPE-19 cells, and mutation of TIMP-1 remarkably weakened the inhibitory effect (Fig. 7B). GnT-V over-expression promoted apoptosis and the production of ROS and MDA, but these effects were inhibited with TIMP-1 mutation (Fig. 7C–E). On the contrary, SOD activity was reduced after GnT-V over-expression but increased with TIMP-1 mutation (Fig. 7F). The expression of gp90^phox^ was increased after GnT-V over-expression but reduced with TIMP-1 mutation. The expression of Nrf2 and NQO1 were decreased after GnT-V over-expression, whereas mutation of TIMP-1 remarkably elevated the expression of Nrf2 and NQO1(Fig. 7G). Western blot and immunofluorescence results showed that GnT-V over-expression increased the expression of GnT-V and VEGF-A, whereas mutation of TIMP-1 only decreased the expression of VEGF-A (Fig. 7H, J). Notably, We observed that N-glycosylation of TIMP-1 was completely promoted by over-expressing GnT-V, but mutation of TIMP-1 reversed the promoting effect (Fig. 7I). This finding indicated that over-expression of GnT-V caused ARPE-19 cells injury by promoting N-glycosylation of TIMP-1.

### GnT-V over-expression promoted RMECs angiogenesis by regulating ARPE-19 cell TIMP-1/VEGF-A axis

**Fig. 8:**
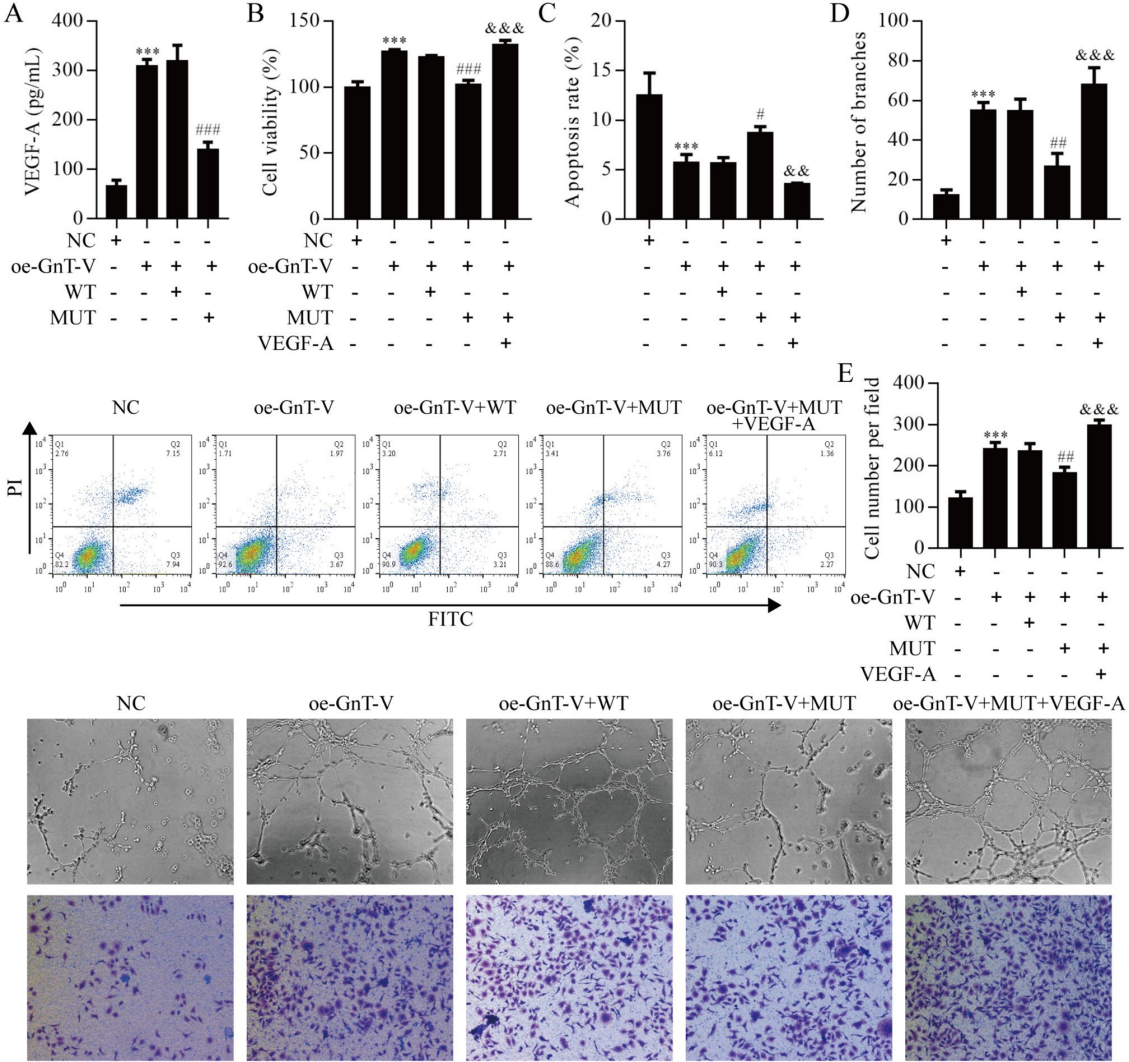
GnT-V over-expression promoted RMECs angiogenesis by regulating ARPE-19 cells TIMP-1/VEGF-A axis. **A** ELISA analysis confirmed the VEGF-A level. **B** RMECs viability was detected by CCK8. **C** Apoptosis of RMECs was detected by flow cytometry. **D** The number of branches. **E** The number of migrating cells. ***P < 0.001 vs. the NC group; ^#^P < 0.05, ^##^P < 0.01 and ^###^P < 0.001 vs. the oe-GnT-V group; ^&&^P < 0.01 and ^&&&^P < 0.001 vs. the oe-GnT-V + MUT group. *ELISA* enzyme linked immunosorbent assays, *NC* negative control, *oe* over-expression, *MUT* mutation, *WT* wild type

The results shown above indicate that VEGF-A is downstream of GnT-V-mediated TIMP-1 N-glycosylation in HG-induced ARPE-19 cells. Next, we elucidated whether ARPE-19 cells release VEGF-A has involved in RMECs angiogenesis. ELISA analysis confirmed the level of VEGF-A in ARPE-19 cell culture medium, we observed that over-expression of GnT-V significantly increased VEGF-A release, but the level of VEGF-A was inhibited by mutation of TIMP-1 (Fig. 8A). The RMECs viability was increased after over-expression of GnT-V but was reversed with TIMP-1 mutation, which was finally boosted by VEGF-A treatment (Fig. 8B). On the contrary, over-expression of GnT-V reduced RMECs apoptosis, which was reversed with TIMP-1 mutation, but apoptosis was terminally repressed by VEGF-A treatment (Fig. 8C). Notably, GnT-V over-expression significantly increased the number of branches and migrating cells but were reversed with TIMP-1 mutation, which were terminally boosted by VEGF-A treatment (Fig. 8D, E). These data suggest that GnT-V over-expression promoted RMECs angiogenesis by regulating ARPE-19 cell TIMP-1/VEGF-A axis.

### Knockdown of GnT-V relieved DR progression

**Fig. 9:**
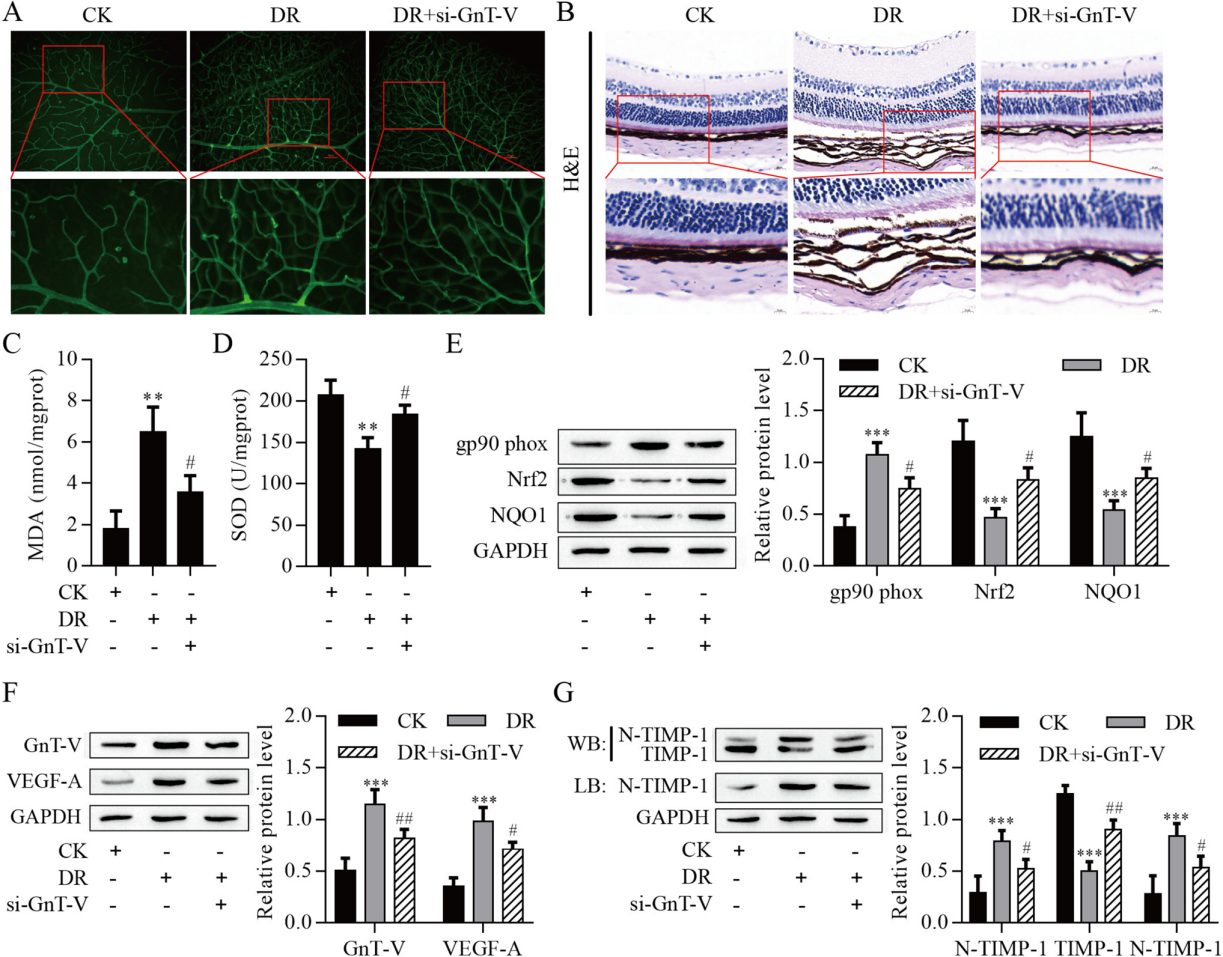
Knockdown of GnT-V relieved DR progression. **A** Representative images of flat-mounted choroid retina. Scale bar = 100 µm. **B** HE staining results. Scale bar = 10 µm. **C** MDA content. **D** SOD activity. **E** Western blot analysis of gp90^phox^, Nrf2 and NQO1 protein levels in retinas. **F** Western blot analysis of GnT-V and VEGF-A protein levels in retinas. **G** Western blot and Lectin blot measured N-glycosylation of TIMP-1. **P < 0.01 and ***P < 0.001 vs. the CK group; ^#^P < 0.05 and ^##^P < 0.01 vs. the DR group. *si* small interfering RNA

Our in vitro studies showed that the GnT-V-mediated TIMP-1 N-glycosylation was important in HG-induced ARPE-19 cells injury and RMECs angiogenesis. Next, we investigated these effects in DR rats. Compared with CK rats, the DR rat retina exhibited retinal may retinal neovascular blebs and choroidal neovascular tufts, which these effects were relieved by knocking GnT-V (Fig. 9A). As shown in H&E staining, compared with CK rats, the DR rat retina exhibited the thickness of RPE cell layer decreased, the structure was loose, and the number of cells decreased, but these effects were reversed with GnT-V knockdown (Fig. 8B). The MDA content was significantly elevated in the DR rats but was reduced in the DR + si-GnT-V rats (Fig. 9C). On the contrary, the SOD activity was significantly reduced in the DR rats, which was reversed with the knockdown of GnT-V (Fig. 9D). Additionally, DR rats significantly increased gp90^phox^ level compared to control retinas and decreased the level of Nrf2 and NQO1, these expressions finally were reversed with knockdown of GnT-V (Fig. 9E). The protein expression of GnT-V and VEGF-A were increased in the DR rats but were decreased in the DR + si-GnT-V rats (Fig. 9F). Notably, We observed that N-glycosylation of TIMP-1 was elevated in the DR rats, but knockdown of GnT-V inhibited TIMP-1 N-glycosylation level (Fig. 9G). Altogether, our data show that HG-mediated GnT-V highly expression promotes VEGF-A release through mediation N-glycosylation of TIMP-1, leading to priming of the RMECs angiogenesis and ARPE-19 cells injury.

## Discussion

Diabetic patients suffer from metabolic disorders due to long-term hyperglycemia. Glucose toxicity is considered to be related to the formation of non-enzymatic glycosylation end products, the activation of polyol pathway and the activation of protein kinase C, but they are not enough to explain the extensive lesions caused by glucose toxicity [12]. Accumulating evidence shows that the occurrence and development of DR is related to the abnormal modification of glycosylation. The study found that 15 glycopeptides from 11 glycoproteins were significantly changed in the serum glycoprotein group of DR patients, including fibronectin (FN1), alpha-2-HS-glycoprotein (AHSG), antithrombin-III (SERPINC1), complement C4-B (C4B), hemopexin (HPX) and inter-alpha-trypsin inhibitor heavy chain H1 (ITIH1), which these glycoproteins are associated with diabetic vascular complications [22]. In this study, we identified 107 N-glycoproteins with 156 N-glycosylation sites (6 up-regulated and 132 down-regulated) abnormal modified proteins in the DR patients. We also determined the abnormal glycoproteomic of FN1, AHSG, SERPINC1, C4B, HPX and ITIH1 in DR vitreous samples. Our present and Ashok et al. previous works have found that the biological functions enriched in the vitreous and serum glycoproteome included positive activation cascade, regulation of protein maturation, and blood coagulation in DR; the cellular components enriched in the blood microparticle, extracellular vesicle and cytoplasmic vesicle; the molecular functions enriched in the glycosaminoglycan binding, serine-type peptidase activity and sulfur compound binding [22]. KEGG signal pathway enrichment analysis results show that the VEGF signaling pathway is the main enrichment pathway, among these N-glycosylation modified differential proteins enriched in VEGF signal pathway, we defined an N-glycosylation of TIMP-1 that plays a critical role in DR progress.

RPE consists of a single layer of hexagonal cells arranged in order, which is located between the retinal sensory cell layer and the Bruch membrane of choroid. RPE layer constitutes the outer barrier of the blood-retinal barrier, which has the functions of barrier, filtration, phagocytosis and secretion [23]. Studies have shown that early DR lesions are related to RPE injury [3, 4]. The main mechanism is that the hyperglycemia environment destroys RPE polar structure, damages the blood-retinal barrier and induces RPE cell apoptosis. One of the most important functions of RPE cells is to selectively transport nutrients and metabolites between the outer layer of retina and choroid. RPE cells secrete a variety of growth factors and cytokines, such as TIMP-1 and VEGF. These factors have the functions of vascular differentiation and vascular permeability regulation [24–26]. Here we found that N-glycosylation of TIMP-1 and VEGF-A levels were elevated in the DR rats and HG-induced ARPE-19 cells.

VEGF signaling pathway is one of the cytokines most closely related to neovascularization in DR. VEGF-A induces angiogenesis and increases vascular permeability by activating VEGF receptor 2 [27]. As the internal barrier of the blood-retinal barrier, RMECs play an important role in the occurrence and development of DR [5]. In our study, TIMP-1 N-glycosylation in the HG environment promoted the secretion of VEGF by ARPE-19 cells. The glycosyltransferase GnT-V, which catalyzes glycosylation modification, also plays a key role [28]. Here we also found that HG-induced increased the level of GnT-V, VEGF and TIMP-1 N-glycosylation. Knockdown of GnT-V can relieve HG-induced ARPE-19 cells injury and DR progression. Studies have shown that the activity and expression of GnT-V in tumors are enhanced, and the abnormal glycosylation of tumor angiogenesis factors or receptors is an indirect factor of angiogenesis [29]. We found that GnT-V-mediated TIMP-1 N-glycosylation promoted VEGF-A expression. On the one hand, glycosylation modification increases the affinity between proteins, prolongs the binding time between VEGF and its receptors, and continuously stimulates the signal pathway of angiogenesis. On the other hand, glycosylation modification indirectly promotes the secretion and release of VEGF-A, enhances the sensitivity of tumor cells to VEGF-A, and causes angiogenesis by changing the function and expression of related factors [30]. Our study also found that HG promoted TIMP-1 N-glycosylation by increasing GnT-V, which induced oxidative stress, apoptosis and VEGF-A expression in RPE cells. Our data support the idea that GnT-V over-expression caused ARPE-19 cells injury by promoting N-glycosylation of TIMP-1.

It was found that RMECs interacted with RPE cells, and the factors and proteins secreted by RPE cells promoted retinal microvascular regeneration by binding with RMECs cell membrane receptors [31]. However, there is no research report on the relationship between protein glycosylation modification in RPE cells promoting VEGF secretion and regulating angiogenesis in DR. We detected up-regulation of glycosylation in the DR rat retinas and HG-induced ARPE-19 cells. Using KIF protected against HG-induced ARPE-19 cells injury by increasing cell viability and inhibiting apoptosis and oxidative stress. More importantly, KIF also remarkably reduced the level of VEGF-A. We observed that TIMP-1 mutation dramatically relieved GnT-V over-expression and HG-induced ARPE-19 cells injury. Notably, over-expression of GnT-V in ARPE-19 cells promoted RMECs angiogenesis by increasing VEGF-A release, and mutation of TIMP-1 inhibited the promoting effect of GnT-V over-expression by inhibited VEGF-A release, which means GnT-V over-expression promoted RMECs angiogenesis by regulating ARPE-19 cells TIMP-1/VEGF-A axis.

In conclusion, GnT-V can promote RMECs angiogenesis and ARPE-19 cells injury through activation VEGF signaling pathway by increasing TIMP-1 N-glycosylation level. Our findings suggest that targeting the GnT-V/TIMP-1 axis might be a promising strategy for preventing DR progression.

## Data Availability

The datasets used and/or analyzed during the current study are available from the corresponding author upon reasonable request.
